# Molecular Targets of Naturopathy in Cancer Research: Bridge to Modern Medicine

**DOI:** 10.3390/nu7010321

**Published:** 2015-01-06

**Authors:** Aamir Ahmad, Kevin R. Ginnebaugh, Yiwei Li, Subhash B. Padhye, Fazlul H. Sarkar

**Affiliations:** 1 Department of Pathology and Oncology, Karmanos Cancer Institute, Wayne State University School of Medicine, Detroit, MI 48201, USA; E-Mails: ahmada@karmanos.org (A.A.); ginneba3@gmail.com (K.R.G.); yiweili@med.wayne.edu (Y.L.); 2 Interdisciplinary Science & Technology Research Academy Department of Chemistry, Maharashtra Cosmopolitan Education Society’s Abeda Inamdar Senior College of Arts, Science and Commerce, Pune 411001, India; E-Mail: bhash46@hotmail.com; 3 Department of Oncology, Karmanos Cancer Institute, Wayne State University School of Medicine, Detroit, MI 48201, USA

**Keywords:** cancer, naturopathy, nutraceuticals

## Abstract

The relevance of naturopathy (defined as the practice of medicine for the treatment of human diseases with natural agents) in human cancer is beginning to be appreciated, as documented by renewed interest in nutraceutical research, the natural anticancer agents of dietary origin. Because of their pleiotropic effects and the ability to modulate multiple signaling pathways, which is a good attribute of natural agents, nutraceuticals have frequently been demonstrated to re-sensitize drug-resistant cancers. The effectiveness of nutraceuticals can be further enhanced if the tools for the relative assessment of their molecular targets are readily available. Such information can be critical for determining their most effective uses. Here, we discuss the anticancer potential of nutraceuticals and the associated challenges that have interfered with their translational potential as a naturopathic approach for the management of cancers. In the years to come, an efficient screening and assessment of molecular targets will be the key to make rapid progress in the area of drug design and discovery, especially focusing on evidence-based development of naturopathy for the treatment of human malignancies.

## 1. Introduction

Cancer is a difficult disease to manage and treat. For the year 2014, it was projected that a total of 1,665,540 new cancer cases will be diagnosed in the United States and approximately 585,720 cancer-related deaths will occur [1]. Although these numbers are frightening, it is interesting to note that the cancer-related mortality has actually declined steadily over last two decades, from 215.1 deaths per 100,000 population in 1991 to 171.8 in 2010 [1]. More than 1.5 million new cases and more than half a million deaths are big numbers that call for sustained efforts in the fight against cancer. The decline in cancer deaths is largely due to the more aggressive screenings and early diagnosis of cancers; but it should not be “mis”-interpreted as evidence of our ability to understand or treat cancer very effectively.

Human cancers represent a large subset of organ-specific subtypes that are often too unique, histologically, as well as genomically. One of the biggest challenges in the effective clinical management of human cancers is the absence of validated therapeutic target(s), especially when evaluating the activity of natural agents (nutraceuticals). This, in turn, has led to a delay in the development of effective targeted therapies. In our fight against cancer, it is imperative to find novel treatment options. Traditionally, the “search” for new treatments has focused on single-targeted agents. Such an approach is based on the reports on the aberrant expression or mutation of a molecular target, implying that the modulation of such a target through a targeted therapy can help to control the growth of tumor, while the fundamentals of cancer biology are typically forgotten, such as that cancer is a heterogeneous disease and the tumor mass contains a heterogeneous population of cancer cells. However, in recent years, it has been realized that such a “single”-targeted therapy might not be the most effective approach. The basis for such a realization is the growing evidence suggesting the switching of cancer cells to alternate survival pathways when confronted with the inhibition of their primary pathway in addition to the presence of a heterogeneous population of cancer cells in a tumor mass. Therefore, a multi-targeted therapy comes across as the most intelligent way of tackling human cancers [2] after many years of denial that multi-targeting would be the rational approach for killing a heterogeneous population of cancer cells in a tumor. As its name suggests, this approach calls for a simultaneous targeting of multiple molecular targets. This can either be accomplished by a combination of a few single-targeted agents or through the use of a single multi-targeting/pleotropic agent (for example, multi-targeting kinase inhibitors). The combination of single-targeted agents often results in increased toxicity [3]. It thus emerges that pleiotropic agents, such as those that occur naturally (by virtue of being natural agents, these agents are largely non-toxic to humans) and that are part of the normal diets in various cultures, the “nutraceuticals”, are promising agents for the treatment of cancer [4,5]. Such an approach defines the importance of “naturopathy” (defined as the practice of medicine for the treatment of human diseases) as a newer field in cancer research and drug development, especially since this field has been ignored for many years. In this article, we will discuss the emerging relevance of naturopathy, the various known molecular targets of nutraceuticals and the assessment of novel targets for naturopathy.

## 2. Naturopathy and Nutraceuticals

Nutraceuticals, or naturally occurring anticancer agents, are pleiotropic in their mode of action [6,7,8,9,10,11,12,13,14,15,16]. As discussed above, cancer is a complex disease that is often a manifestation of multiple deregulated pathways where cancer cell heterogeneity is the main cause of treatment failure in modern medicine. Therefore, with their ability to regulate multiple molecular targets, nutraceuticals in a naturopathic approach stand out as ideal candidates for inhibiting tumorigenesis and/or for achieving better treatment outcome in patients diagnosed with cancers. The single-targeted agents often fail in clinical trials [17]. Even when they show promise in clinical trials and get approved for use in the clinic, their sustained use leads to the development of resistance, for which there is no curative treatment. Such drug-resistant cancers are much more aggressive and difficult to manage and remain one of the leading causes of cancer-associated morbidity and mortality.

An efficient multi-targeted therapy can be as simple as combining inhibitors of closely-related pathways, like, for example, kinase inhibitors [18]. It can be envisioned that cancer cells switch their dependency on closely-related signaling in the case of the suppression of their primary signaling pathway, collectively known as redundant cell signaling pathways. Such switching to closely-related alternates is easily accomplished, as opposed to a signaling pathway that is very distinctly related. Thus, simultaneous inhibition of closely-related signaling pathways is often the first line of research investigation. However, the use of single-targeted agents has also revealed some more complex scenarios. For example, aromatase inhibitors are the primary therapy for breast cancer with overexpression of estrogen receptors (ERs) [19], but it has been reported that resistance to aromatase inhibitors can involve the activation of a very distinct signaling, the one mediated by HER2 [20]. It is interesting to note that signaling through ERs and HER2 (the tyrosine kinase receptor belonging to the epidermal growth factor receptor family) represents two major subtypes of breast cancer. This report [20] reveals that suppression of one prominent breast cancer signaling is overcome by tumor cells by activating an alternate prominent signaling. It was also reported that HER2 is a negative regulator of ER, and therefore, when HER2 signaling was blocked via targeted antibody, ER expression goes up, and the aromatase inhibitors could once again become effective.

The study of the mechanism of aromatase inhibitor resistance discussed above [20] teaches us a few fundamental lessons. First, the inhibition of one major cellular signaling can result in the activation of an alternate major signaling. Second, there is an immense regulation of cellular signaling in physiological systems where signaling pathways mutually regulate each other through an overwhelming cross-talk. HER2 negatively regulates ER, and that is why the authors noted the re-expression of ER when HER2 was blocked. There is strong evidence of extensive crosstalk between ER and HER2 signaling pathways [21], which means that the ER, in turn, can negatively regulate HER2. Collectively, this seems to imply that suppression of ER in ER-dependent tumors can lead to drug-resistance through activation or de-repression of HER2 signaling, while, on the contrary, suppression of HER2 in HER2 over-expressing breast cancers can result in resistance to HER2-targeted therapies through activation or de-repression of ER signaling. This finding, together with many other examples, clearly suggests that a rational approach for combining multiple agents must be scientifically tested for the treatment of human malignancies.

In order to test whether nutraceuticals can be beneficial in the above scenario because of their pleiotropic nature, we looked for the reports on the effects of some very well-studied nutraceuticals, such as curcumin, resveratrol and 3,3′-diiindolylmethane (DIM), on ER and HER2 signaling pathways. Indeed, there is evidence for the modulation of ER signaling [22,23,24,25,26], as well as HER2 signaling [27,28,29,30] by curcumin, resveratrol and DIM. These three are not the only nutraceuticals with anticancer potential, but this quick search did add to the argument that such agents might be beneficial against ER and HER2 signaling pathways. It should be mentioned that the studies cited here have looked individually at either ER or the HER2 signaling pathways. Logically, it might make sense to expect an action of these agents against the two signaling pathways within the same cells, as well.

There is not enough data to either prove or disprove such pleiotropic action of nutraceuticals against ER and HER2 signaling. However, that is not the main point of discussion. Through the examples of ER and HER2 signaling, it becomes evident that the suppression of one signaling pathway leads to the adaptation of cancer cells to an alternate pathway. Considering the growing importance of more signaling pathways, such as PI3K/Akt, NF-κB, Notch, Wnt, hedgehog, *etc.*, which are relevant to cancer progression, thus, the cross-talk becomes more extensive. The choice of cancer cells to find an alternate pathway also increases, and now, it might be argued that targeting of one, two or even more signaling pathways may still not be the best strategy to suppress tumor growth, while such as a strategy will only increase unwanted toxicity, which will kill the patients before realizing their treatment benefit. Clearly, there are so many signaling pathways for the cancer cells to fall back on, and for that reason, a “systems biology” approach for identifying the most important set of oncogenic pathway is becoming a new era of modern medicine. Again, pleiotropic agents, such as nutraceuticals, are our best option, because of their reported activity against virtually every single cancer-relevant signaling pathway [31,32,33,34,35,36,37], but most importantly, for their attributes as non-toxic agents. Therefore, naturopathy would likely become a new arsenal for combating the fight against cancers.

## 3. Molecular Targets of Nutraceuticals and Their Cytotoxic Effects

It is quite evident from the discussion above that nutraceuticals are multi-targeting agents. They modulate an array of signaling pathways, as well as individual molecular targets, and a discussion on these is beyond the scope of this article. The available literature reveals that natural anticancer agents have been shown to touch upon virtually every single molecular target. Just to point out a few major signaling pathways/targets affected by nutraceuticals, we can identify EGFR family receptors, Ras/Raf signaling, MAPK/ERK pathway, PI3K/Akt/mTOR pathway, Notch family, Wnt/β-catenin signaling, Sonic hedgehog signaling, hormone receptors (such as ER/progesterone receptor), TGF-β signaling, insulin-like growth factor signaling, cAMP signaling, the STAT3 signaling pathway, *etc.* In addition to these classical targets, nutraceuticals are also being realized to efficiently modulate emerging targets, such as cancer stem cells [38,39,40], microRNAs (miRNAs) [39,41,42,43], epithelial-mesenchymal transition (EMT) [44,45] and the causes of epigenetic modifications [46,47].

Through their action against these molecular targets, nutraceuticals attack the cancer cells at many different levels: they inhibit cancer cells’ proliferation, induce apoptosis/cell cycle arrest and suppress invasion/metastasis/angiogenesis. These cytotoxic effects are mediated through the action of nutraceuticals against factors, such as bcl2, survivin, vascular endothelial growth factor (VEGF), matrix metalloproteinases (MMPs), urokinase-like plasminogen activator (uPA), *etc.* In addition to numerous reports on the *in vitro* effects of nutraceuticals, there are many *in vivo* reports that document the beneficial anticancer effects of nutraceuticals in animal model systems; however, control and rationally-designed phase II/III clinical trials are awaited, although some early clinical trials are beginning to show some promising results.

## 4. Nutraceuticals as Anticancer Agents: Challenges

**Figure 1:**
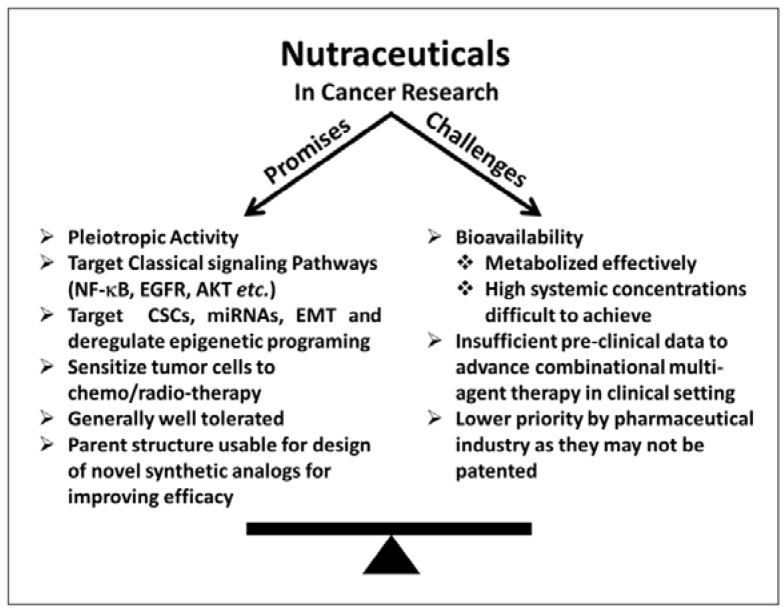
The importance of nutraceuticals as anticancer agents is increasingly being recognized. They hold a lot of promise, as evidenced by multiple reports on their ability to modulate key signaling pathways/molecules that influence tumorigenicity. However, some challenges, particularly their bioavailability, have hindered their progress through clinical trials. In order to realize the true potential of nutraceuticals as anticancer agents, the challenges need to be overcome.

With all the available data, it appears that naturally-occurring anticancer agents are well placed to be employed in the clinical setting for the treatment of human cancers. However, this has not yet happened in the classical manner of clinical trials (Figure 1). There are few reasons that have obstructed the clinical development of natural compounds as anticancer agents. The first and foremost reason is the issue of bioavailability. Study after study has reported that nutraceuticals demonstrate poor bioavailability, when assessed in pharmacological studies. While this is a major road block, it is important to understand that these agents are part of the normal human diet, and as part of natural diets, they are absorbed and processed very efficiently physiologically with sustained low levels, which may be highly important for rendering their biological activity. This also means that they need to be effective within the short window ranging from minutes to few hours when they are detectable in circulation. This is not an ideal scenario according to conventional pharmacology, and thus, such poor bioavailability gets in the way of their effectiveness as anticancer agents in diseased subjects, as viewed through the lens of classical clinical trials with pharmacological agents. In order to replicate the *in vitro* effects, these nutraceuticals should persist in circulation for longer durations. To partially overcome this bioavailability issue, use of higher doses has been recommended and evaluated. This has led to the second challenge, the issue of higher doses-associated unwanted toxicity, even though they are natural agents. As with any drug, even natural agents can be tolerated up to an extent, and once used at very higher doses, toxic side effects become more evident.

Does this mean that all of the promising preliminary anticancer activity of nutraceuticals can never be translated into clinical reality? The answer might not be very straightforward, but efforts are underway to make sure that such a promise does not go to waste. One strategy to overcome limited bioavailability is the use of novel formulations that ensure better and sustained systemic release [48,49,50,51,52,53,54,55,56]. Such formulations have resulted in better efficacy and need to be tested further in clinical studies. Another approach is the synthesis of novel analogs of nutraceuticals. A number of analogs of various nutraceuticals have been reported [40,57,58,59,60,61,62,63,64,65,66,67,68,69,70,71,72,73,74]. This is another effective strategy to improve the efficacy of nutraceuticals by chemically modifying the structure of the compound, leading to enhanced cytotoxicity, as evidenced by lowered IC_50_ values. Although it sounds simple, this is a tedious process that involves the synthesis of many putative chemical analogs, keeping intact the main chemical moiety. Synthesis is then followed by screening of compounds for their anticancer activity. Our own laboratory has been interested in addressing the bioavailability issue of nutraceutical curcumin through the synthesis of novel analogs. Our efforts have led to the synthesis of difluorinated curcumin (CDF), which has demonstrated better efficacy as an anticancer agent with enhanced bioavailability [75,76,77,78,79,80,81]. However, this compound needs further clinical development for the treatment of human malignancies.

## 5. NF-κB Signaling: The Master Pathway Regulated by Nutraceuticals

NF-κB (nuclear factor-kappa B) is a crucial signaling pathway involved in the progression of human cancers [82,83,84,85]. NF-κB pathway includes several important molecules, such as NF-κB and IκB Kinase (IKK); however, NF-κB is the key protein in the pathway that has been extensively implicated as a promising target for cancer therapy without much success. NF-κB is a transcription factor that exists in a latent state in the cytoplasm bound to IκB inhibitory proteins. Pro-survival stimuli result in IKK-dependent phosphorylation and subsequent proteasome-mediated degradation of the inhibitory IκB proteins. This results in the release of NF-κB, which migrates to the nuclear compartment and regulates the transcription of multiple target genes. These target genes are largely involved in the regulation of cell proliferation, invasion and metastasis. Because of such a prominent and central role of NF-κB, its inhibition by nutraceuticals is considered good proof in support of their anticancer ability. Factors, such as VEGF, MMPs and uPA, are all influenced by NF-κB signaling, thus providing a direct connection between NF-κB signaling and an aggressive phenotype, and further provide a proof-of-concept that naturopathy could be a novel strategy in modern medicine.

The relevance of NF-κB signaling in cancer progression is further confirmed by the fact that this signaling pathway is probably the most studied pathway when it comes to assessing the activity of potential anticancer agents. Often, this is the very first pathway evaluated. As a consequence, almost every single nutraceutical has been documented to inhibit the NF-κB signaling pathway to some extent. In particular, there is overwhelming data supporting the inhibition of NF-κB signaling by curcumin [86,87,88,89], which almost makes it appear as if curcumin is a specific inhibitor of NF-κB signaling; however, the benefit of curcumin is limited due, in part, for its poor systemic and target tissue bioavailability. As discussed above, curcumin failed in translational studies because of its poor bioavailability, and thus, our novel synthetic analog (CDF) is perhaps a successful attempt for improving the bioavailability of an active natural compound. Our initial experiments revealed that the levels of CDF were 10-times higher than curcumin in the pancreas of mice [80]. Based on these observations, it is safe to conclude that the enhanced anti-tumor activity of CDF is in part due to its potent inhibition of NF-κB signaling and that the activity in the screening assay is also consistent with the pharmacokinetic results, as discussed above. In addition to curcumin and CDF, a number of other nutraceuticals target NF-κB, which forms the basis for their anticancer activity [74,90,91,92,93,94,95].

## 6. Conclusions and Perspectives

Cancer research has come a long way from the time when searching for single-targeting agents was the norm in the field of drug discovery. It is now widely accepted that cancer is an even more complicated disease than ever envisioned, and a tumor mass is composed of a highly heterogeneous population of cancer cells having aberrations in distinct, yet multiple sets of genes. The tumor cell heterogeneity together with intrinsic (*de novo*) and extrinsic (acquired) drug resistance appear to be the key reasons for the treatment failure of conventional therapeutics. Therefore, a better understanding of drug resistance phenotype is the current area of research, which has made us realize that the inhibition of the major signaling pathway often leads to switching of cancer cells to utilize alternate pathways for their survival and, thus, resist therapeutic benefit. All of this knowledge has led to advocating the use of combinational therapies or the use of pleiotropic agents, such as naturally occurring anticancer agents of dietary origin, to achieve better treatment outcomes for patients diagnosed with cancers. To that end, nutraceuticals have shown great promise in *in vitro* studies, but have fallen short in translational studies. The bioavailability of nutraceuticals remains a major concern. One way of overcoming this issue is through the synthesis of novel analogs of established nutraceuticals. A number of novel nutraceuticals are under investigation in our laboratory and others throughout the world, and thus, quick and efficient screening of new and existing compounds will be the key to finding chemical structure(s) that can be used in the future for the clinical management of human cancers, which clearly support naturopathy as a tool for modern medicine. In addition to the classical signaling pathways, new screening tools will be helpful in evaluating emerging molecular targets, such as microRNAs, and target gene expression modulated by nutraceuticals. This is a step in the right direction that integrates emerging techniques with state-of-the-art knowledge. Combining this further with computational tools, such as modeling/docking studies, bioinformatics and systems biology, should be the goal in moving forward the field of naturopathy in modern medicine. 
